# Psychiatry

**Published:** 1905-10-07

**Authors:** 


(*To be continued.*)

### PSYCHIATRY.

(*Continued from page* 343.)

**Amentia or Mental Defect.**—Dr. Joseph Shaw Bolton,[4] of Rainhill Asylum, Lancashire, publishes a valuable contribution to the symptoms, clinical and pathological, of amentia. The term is defined by the scope it occupies in mental pathology. It includes "not only idiots and imbeciles, but also a large number of cases which exhibit a slighter degree of cerebral agenesis and deficiency known as "feeble-mindedness," while the subjects of the same are also sometimes called "mental defectives." Dr. Bolton states that cases of amentia as thus described and included exhibit "physical stigmata of degeneracy," *e.g.* abnormal forms of the skull, face, ears, teeth, palate, hair, mammæ, and palpebral fissures more frequently in greater severity than do normal individuals or the higher types of mental disease. They more frequently have an heredity of mental alienation, "and not uncommonly two or more members of the same family may be found in the same institution." These are also the cases about whom, apart from purely domestic reasons, the friends are never tired of instituting inquiries or expressing desires for their discharge, and it is among the relatives of these patients that the major portion of "borderland" cases of mental disease are found. Many of the milder cases of idiocy and imbecility are in asylums, because they are so unstable that they cannot be kept outside asylums for any long period. They are slow to develop extreme and apathetic dementia. Such cases are, however, more prone to dementia at certain critical epochs of life, *e.g.*, at the period of puberty and adolescence (ages of 18 to 25 in males, and of 16 to 21 in females), or at periods of pregnancy and parturition, especially if the complicating factor of illegitimacy is present. At any one of these or similar critical periods, as, *e.g.*, the menopause and the period of presenility the degenerate may fail to respond to the demands of what is, to his normal brother, a normal environment and may reveal his or her previously latent mental defect more conspicuously. The mere age of mental breakdown is itself of little importance. As regards the emotional tone of the ego, as manifested in similar breakdowns, the importance of the said emotional tone is slight. Temperaments of melancholic or joyous nature are deeply founded in the lower ego. Some idiots and imbeciles may develop depressive delusions of a religious character, or delusions of being poisoned, but such cases are distinctly rare. Dr. Bolton studied 728 cases and found them to be of the following types:—

| | Males | Females | Total |
|---|---|---|---|
| 1. Idiocy and imbecility (primary and secondary) ... ... | 51 | 43 | 94 |
| 2. Excited cases, and cases of moral idiocy and imbecility... ... | 22 | 64 | 86 |
| 3. Recurrent cases... ... ... | 17 | 30 | 47 |
| 4. Hysterical cases... ... ... | 0 | 6 | 6 |
| 5. Epileptic idiocy and imbecility... | 6 | 8 | 14 |
| 6. Cases with systematised delusions, including paranoia ... | 10 | 16 | 26 |
| Total mental defects ... | 106 | 177 | 283 |

Dr. Bolton also classifies his cases according to the grade of development of intelligence into the following:—

(*a*) Extreme mental deficiency (idiocy and low-grade imbecility), with epilepsy.

(*b*) Extreme mental deficiency (idiocy and low-grade imbecility), without epilepsy.

(*c*) Slight mental deficiency, with epilepsy.

(*d*) Slight mental deficiency, without epilepsy.

Many typical symptoms and illustrative cases are given of each class. Among "cranks" and asylum "curiosities" he describes types possessing fairly constant characters. "They are extremely vain, conceited, and grandiose, and frequently form the 'show-birds' of asylums. Their general behaviour is grotesque and usually amusing or absurd, and their actions are uncertain, erratic, and frequently weird. They are usually fond of finery, and they wear absurd and curious decorations, to which they often attach a symbolic meaning. . . . For similar reasons they at times perform strange actions—*e.g.* constantly holding a finger in one place . . . . going out of their way to touch articles as they pass, etc." Sometimes they are artistic, but usually their work is grotesque and even incoherent. These characteristics find their counterpart and cause in their association of ideas, which is usually erratic and frequently grotesque. Some cases are solitary in their habits and peculiar in their behaviour and are given to morbid introspection; and such patients at times invent some new system of morbid philosophy. They readjust the world on lines of their own, and invent all kinds of new words to express either directly or in a kind of "shorthand" their meaning. It may be added that these patients as a class (like other types of idiots or imbeciles) only differ from certain "sane" individuals in the absurd and grotesque extremes to which they carry their ideas and their resulting behaviour and actions, and that their eccentricity and "stereotypism" in conduct also only differ in degree from the vagaries of conduct often seen among "cranks" in society. Of seventeen patients of the "crank" and "asylum curiosity" class, concludes Dr. Bolton, a considerable proportion are good workers. Thus of seven males four were good workers and one did a little work. Of ten females five were good or fair workers and one did a little work.

[4] Jour. of Ment. Sci., July 1905.

